# Craniofacial size in individuals with supernumerary teeth: A multicenter geometric morphometric study

**DOI:** 10.1186/s12903-026-09159-9

**Published:** 2026-07-07

**Authors:** Sofia Bakirtzi, Eva Henninger, Luca Friedli, Georgios Kanavakis, Nikolaos Gkantidis

**Affiliations:** 1 Department of Orthodontics and Dentofacial Orthopedics, University of Bern, Bern, 3010 Switzerland; 2 Department of Orthodontics, School of Dentistry, National and Kapodistrian University of Athens, Athens, 11527 Greece; 3 Department of Pediatric Oral Health and Orthodontics, UZB-University Center for Dental Medicine, University of Basel, Basel, Switzerland

**Keywords:** Odontogenesis, Permanent dentition, Supernumerary tooth, Craniofacial development, Tooth agenesis

## Abstract

**Background:**

Although tooth agenesis has been consistently associated with reduced craniofacial size, the craniofacial implications of supernumerary teeth remain unexplored. This study aimed to assess whether the presence, number, and location of supernumerary teeth are associated with craniofacial size in non-syndromic individuals, and whether these effects differ by sex.

**Methods:**

A retrospective multicenter study was performed using > 10,000 orthodontic records from Switzerland and Greece (2002–2023). The sample included 200 individuals with supernumerary teeth (125 males, 75 females; mean age 12.7 ± 5.7 years) and 200 age- and sex-matched controls. Supernumerary teeth were classified by number and location (maxilla vs. mandible). Craniofacial morphology was described on pre-treatment lateral cephalograms using 117 landmarks and semi-landmarks across different anatomical regions. The size of these regions and of the entire craniofacial complex was measured as the natural logarithm of centroid size (ln[CS]). Multiple linear regression models were applied to test associations between craniofacial size, sex, age, and supernumerary tooth number or location. Statistical significance was set at α = 0.05.

**Results:**

Age and sex were strong predictors of craniofacial size (*P* < 0.001), with larger dimensions in males and older individuals. The number of supernumerary teeth showed a small positive association with maxillary size (β = 0.011; *P* < 0.005), evident only when rare severe cases (> 2 teeth) were included, and no significant effects on the cranial base, mandible, or overall facial size. Location-specific analyses indicated that individuals with mandibular supernumerary teeth (*n* = 50) had larger mandibular (β = 0.016; *P* = 0.009) and maxillary dimensions (β = 0.022; *P* < 0.001), with males also showing slightly larger overall facial size (+ 2.5%; *P* = 0.014). Maxillary supernumerary teeth (*n* = 145) showed no significant craniofacial size differences.

**Conclusion:**

Supernumerary teeth exert limited, region-specific effects on craniofacial size. While overall facial dimensions remain largely unchanged, mandibular supernumeraries, though uncommon, are associated with modest enlargement of both jaws in males. These findings contrast with the consistently reduced craniofacial size in cases with tooth agenesis, underscoring the differential developmental impact of different tooth number anomalies.

**Supplementary Information:**

The online version contains supplementary material available at https://doi.org/10.1186/s12903-026-09159-9.

## Introduction

Supernumerary teeth, or hyperdontia, are an odontogenic anomaly characterized by the presence of an excessive number of teeth relative to normal dentition, with an estimated prevalence of approximately 4.6% of the population [1, 2]. Their prevalence differs across populations, being more common in Asian than in White populations. They may erupt, but frequently remain unerupted [1, 3, 4]. This condition is more common in males than females, with a reported ratio of 2:1, and most cases are idiopathic. It is less frequent in the deciduous dentition (0.3–0.8%) compared to the permanent dentition [1, 2].

Supernumerary teeth may occur unilaterally or bilaterally and can present as a single tooth or multiple teeth, with higher prevalence as single teeth and in the maxilla [1, 2]. Morphologically, supernumerary teeth may appear as normal teeth, conical, tuberculate, or as odontomas [3, 5]. The most common location is the anterior maxilla, particularly between the central incisors, where supernumerary teeth are referred to as mesiodens. Single mesiodens account for approximately 30% of cases and often present in a conical or hypoplastic form. Multiple supernumerary teeth are more frequently observed in the maxillary premolar region. In the molar regions, supernumerary teeth are termed paramolars or distomolars depending on their position [1, 5].

Supernumerary teeth can cause a range of complications, including impaction or ectopic eruption of permanent teeth, rotation or displacement of adjacent teeth, crowding, and malocclusion. They may also contribute to dilacerations, root resorption, delayed or abnormal root development, or to the formation of fistulas and cystic lesions [2, 3, 5–8]. These potential consequences highlight the importance of early diagnosis and timely management.

Supernumerary teeth can appear in both syndromic and non-syndromic individuals, with higher prevalence among syndromic cases. Well-documented associations include cleidocranial dysplasia and familial adenomatous polyposis (FAP), as well as Down syndrome, Nance-Horan syndrome, trichorhinophalangeal syndrome type I, Rubinstein-Taybi syndrome, Opitz G/BBB syndrome, oculofaciocardiodental syndrome, and Robinow syndrome [5, 9, 10].

The etiology of supernumerary teeth is multifactorial, involving both genetic and environmental influences. Although most cases are idiopathic [5, 9], both autosomal dominant (OMIM 108700), autosomal recessive, and X-linked non-syndromic forms have been reported [11, 12]. Multiple supernumerary teeth are uncommon and more often associated with syndromes [8–11]. Several theories have been proposed, including atavism, tooth bud dichotomy, hereditary mechanisms, progress zone theory, and unified models. However, hyperactivity of the dental lamina, resulting from proliferation of residual epithelial cells, is currently the most widely accepted mechanism, likely triggered by disturbances during embryogenesis [3, 7, 10]. Mutations in genes such as APC, RUNX2, and FAM20A have been implicated, and disruptions in major developmental signaling pathways (Wnt, SHH, BMP, FGF, and EDA) can alter odontogenic potential, shifting normally inactive tissues toward tooth formation and resulting in additional teeth [10].

Many genes implicated in supernumerary teeth also participate in craniofacial morphogenesis, suggesting shared developmental pathways [10]. Evolutionary perspectives further support a common mechanism for tooth and craniofacial development [13–16]. Tooth agenesis has been consistently associated with craniofacial shape alterations [17–19], as well as with reduced facial size [20, 21], likely reflecting early disturbances in signaling pathways that influence both dental and craniofacial growth. However, the relationship between supernumerary teeth and craniofacial form remains unknown. Determining whether an excess of teeth corresponds to increased craniofacial size would provide insight into whether dental and craniofacial development are coordinated in a manner analogous to tooth agenesis, which is consistently associated with reduced craniofacial dimensions [20, 21]. Both types of tooth-number anomalies are associated with generalized alterations in tooth size across the dentition [22]. Importantly, the well-established association between tooth agenesis and reduced craniofacial size in modern humans [20, 21] is consistent with evolutionary mechanisms that have driven gradual facial and tooth reduction across hominin evolution [13–16]. If supernumerary teeth do not relate to larger craniofacial dimensions, this would support evolutionary trends toward facial reduction that continue in modern humans, suggesting that supernumerary tooth formation arises from more localized developmental mechanisms. Therefore, the aim of this study was to examine the association between supernumerary teeth formation and craniofacial size.

## Materials and methods

The material for this study was primarily derived from a sample that has been previously described in a study investigating supernumerary tooth patterns in non-syndromic individuals [1]. Relevant methodological details are summarized here to facilitate comprehension of the present study. 

### Ethical Approval

Ethical approval was obtained from the Research Ethics Committees of the cantons of Bern, Neuchâtel, Basel, and Jura, Switzerland (Protocol No. 2022 − 00399, approved on 20 June 2022), as well as from the Institutional Ethics Committees of the Dental Schools of the National and Kapodistrian University of Athens (Protocol No. 518/05.09.2022, approved on 13 October 2022) and the Aristotle University of Thessaloniki, Greece (Protocol No. 182/10.02.2023, approved on 16 March 2023).

All procedures were conducted in accordance with relevant guidelines and regulations. Written informed consent was obtained from all participants prior to their inclusion in the study. For participants who were minors, written informed consent was obtained from their parents or legal guardians in accordance with applicable national regulations.

### Study design and data collection

This retrospective study involved the analysis of patient records from the Department of Orthodontics at the University of Bern, the University of Basel, two private orthodontic practices in Switzerland, the National and Kapodistrian University of Athens, and the Aristotle University of Thessaloniki. A total of more than 10,000 archived patient files were reviewed, covering various time periods between 2002 and 2023, depending on the institution.

At each location, researchers systematically screened the archives, including medical and dental histories, intraoral and extraoral photographs, and radiographs. Individuals who met the inclusion criteria were identified, and their data were anonymized. For each selected case, the available pre-treatment panoramic and cephalometric radiographs were examined. Additional diagnostic records such as dental models, intraoral and extraoral photographs, and medical/dental histories were also reviewed to support diagnostic accuracy. These records represent standard documentation for all patients undergoing orthodontic treatment.

Participants with supernumerary teeth were selected based on the following criteria [1]:

- Individuals between 8 and 50 years of age at the time of pretreatment radiographs. For individuals younger than 12 years at the time of the initial radiograph, any subsequent radiographs obtained at older ages were also reviewed to confirm the absence of late-developing supernumerary teeth (none were detected).
- Individuals of European ancestry (White), due to the predominance of this racial group in the available archives. Other racial backgrounds were underrepresented and were thus insufficient to form statistically meaningful comparison groups.
- At least one supernumerary tooth.
- No diagnosed syndromes, systemic diseases, or other conditions affecting craniofacial development.
- Absence of extensive dental restorations that could affect craniofacial form or interfere with radiographic assessment of craniofacial structures or supernumerary teeth.
- Sufficient-quality panoramic and cephalometric radiographs, or cone-beam computed tomography (CBCT) scans.
- No history of orthodontic treatment or other interventions that could influence craniofacial morphology.
- Absence of additional dental anomalies (excluding third molars).

**Fig. 1:**
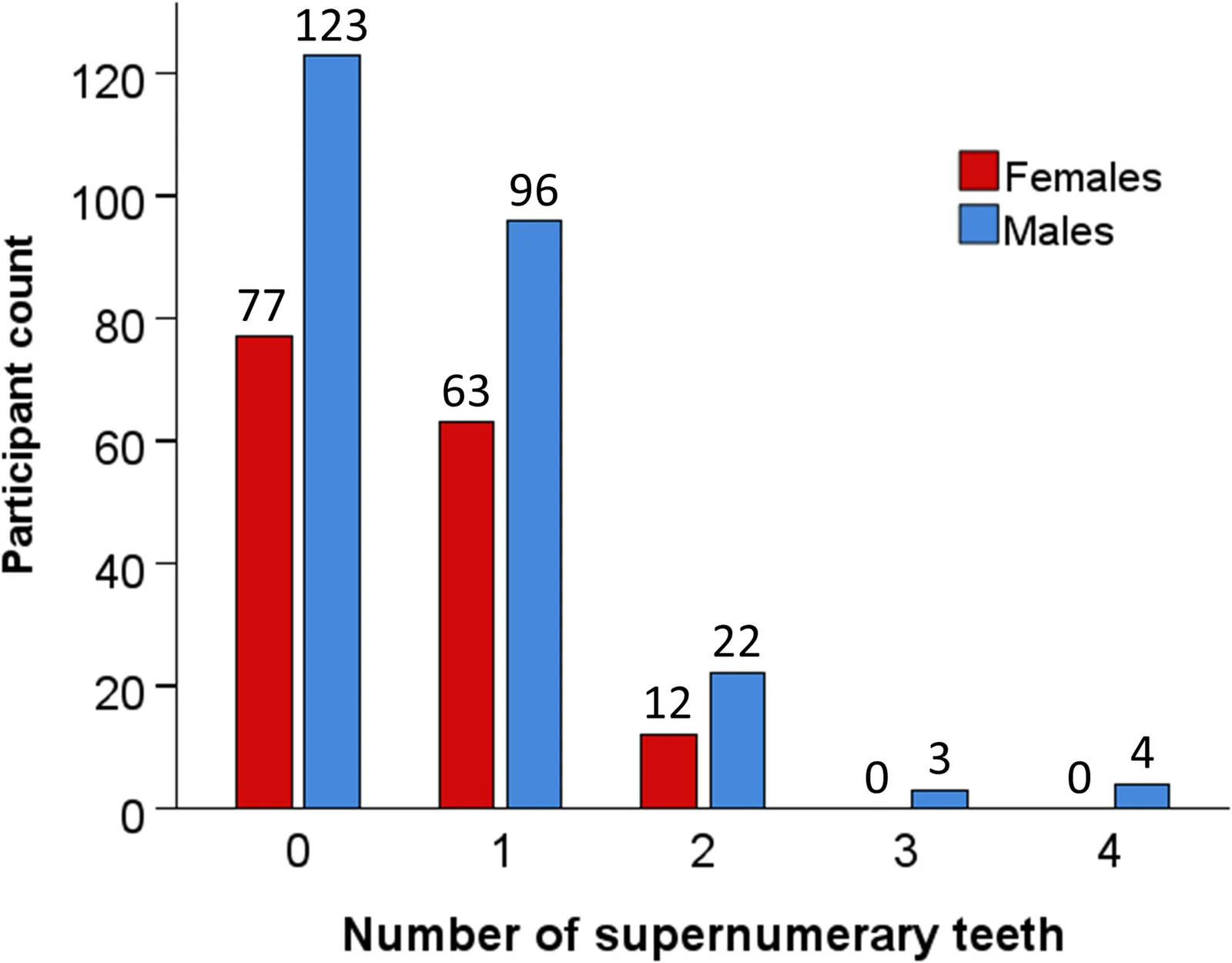
Bar graph depicting the supernumerary teeth frequency distribution per sex in the entire sample

Patient records were thoroughly reviewed, including medical and dental histories, intraoral and extraoral photographs, and radiographic images. The tooth sequence of each subject was identified on panoramic radiographs and recorded in a Microsoft Excel spreadsheet (Microsoft Corporation, Redmond, WA, USA), along with the patient’s age and sex. The final sample consisted of 200 individuals, 75 females (mean age: 13.1 ± 7.8 years) and 125 males (mean age: 12.1 ± 4.5 years), with 90 and 168 supernumerary teeth, respectively. The distribution of age, sex, and number of supernumerary teeth is illustrated in Additional File 1: Figure S1, revealing that age distribution spans a broad range for both sexes. The relatively flat LOESS curves indicate minimal variation in age with increasing numbers of supernumerary teeth. Approximately 80% had a single supernumerary tooth, while around 15% presented with two. Although supernumerary teeth were more frequently observed in males and only a few males showed more than two supernumerary teeth, no significant sex-based differences were observed in the severity of the condition (Fig. 1). These data are in line with previous findings on supernumerary tooth patterns in non-syndromic White European populations [1].

For each participant with supernumerary teeth, one control subject was selected on a 1:1 basis. Controls were matched by sex and age within a margin of ± 6 months and were required to have a full dentition, excluding third molars. All control subjects were retrieved from the same archives as the corresponding cases, using a consecutive search approach.

The total sample size of 400 individuals was determined based on case availability and prior empirical evidence and is considered adequate for the study’s objectives. In geometric morphometric research, sample size cannot be determined solely through conventional statistical formulas [23, 24]. In this study, the primary subgroups, namely males and females without or with one supernumerary tooth, included at least 60 participants each. This number was considered sufficient to detect meaningful differences [20, 21, 23, 24].

### Outcome measures

Information on craniofacial size was obtained through the identification of anatomical landmarks on lateral cephalometric radiographs using Viewbox 4 software (dHAL Software, Kifissia, Greece). Morphometric analyses were performed on the same software, with customized settings tailored to the specific requirements of the study [25]. The primary outcomes of this study were the sizes of the following anatomical structures: the overall craniofacial configuration (excluding the superior and posterior parts of the cranium, due to inconsistent radiographic coverage and their relative distance from the primary region of interest, namely the facial structures), the cranial base, the maxilla, and the mandible. Cephalograms were scaled to actual size using the reference ruler available during image acquisition.

**Fig. 2:**
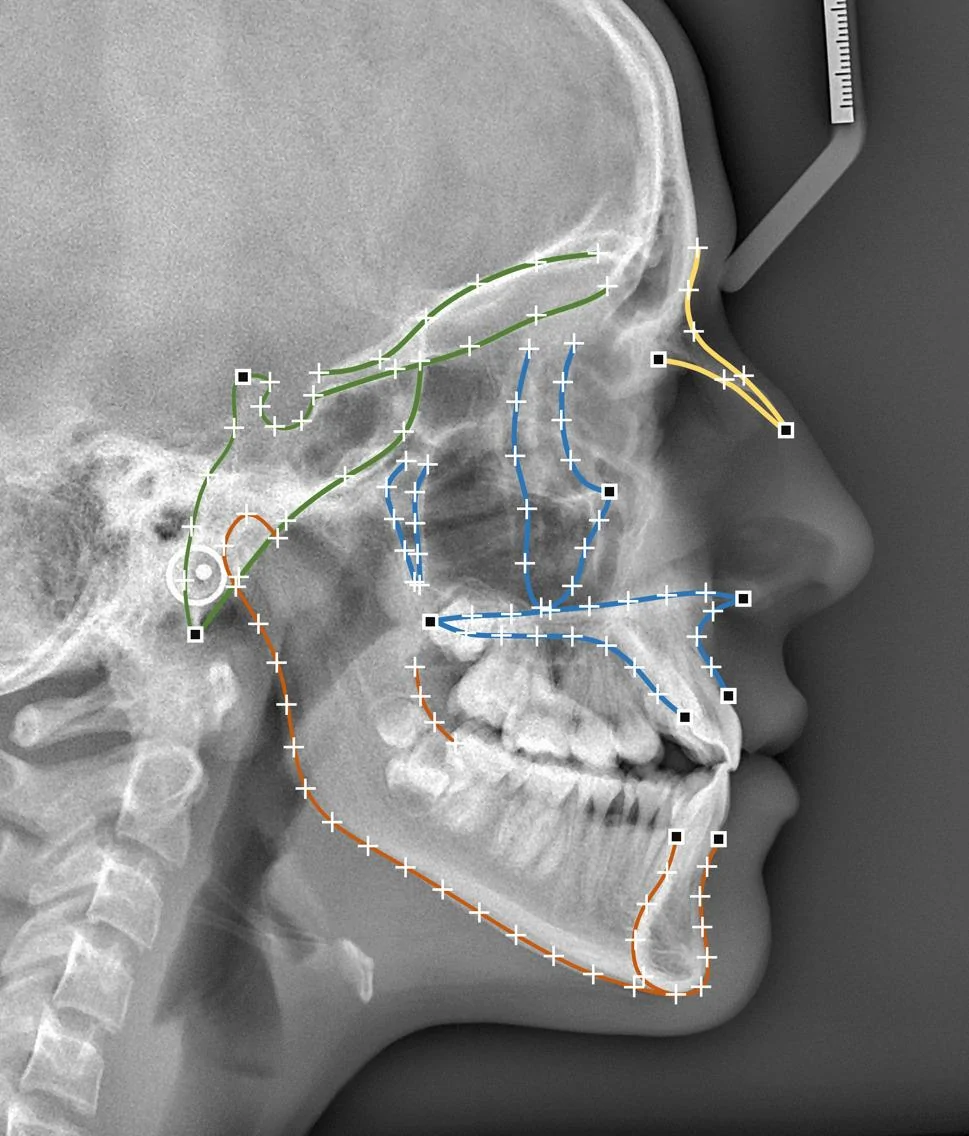
Cephalometric radiograph of a female individual with a supernumerary mandibular right third molar, illustrating the landmarks and semilandmarks used to characterize craniofacial morphology and associated curves. The green line represents the cranial base, the blue line the maxilla, the orange line the mandible, and all lines together define the entire craniofacial configuration. Fixed landmarks are indicated by black squares, and semilandmarks by white crosses

According to a previously published protocol [20], fifteen anatomical curves were used to comprehensively capture craniofacial skeletal morphology, defined by a total of 117 landmarks. Of these, 11 were fixed landmarks, identified based on clear anatomical features (e.g., anterior nasal spine [ANS], posterior nasal spine [PNS]) or defined as endpoints of curves. The remaining 106 were semi-landmarks [26], initially placed at equidistant intervals along these curves, which were allowed to slide along their respective curves (Fig. 2). The ten most superior semi-landmarks in the frontal region previously included [20] (Frontal 1–5, Internal frontal, Internal frontal 1–3, Ethmoid 5) were excluded from the present analysis because certain radiographs did not depict those anatomical areas. Due to the absence of anatomical homology between semi-landmarks among different samples, semi-landmarks were allowed to slide along their respective curves describing the anatomical structures [27]. This process was repeated three times in order to minimize bending energy between each shape configuration and a reference configuration, corresponding to the average shape configuration of the sample. At the end of this iterative process, maximum possible homology was achieved between semi-landmarks. Semi-landmarks can be slid by minimizing either Procrustes distance or bending energy. We opted for bending-energy minimization because it constrains semi-landmark displacement according to the deformation of the surrounding configuration, thereby better preserving local anatomical relationships and resulting in smoother, more biologically meaningful shape changes. In contrast, Procrustes-distance minimization moves semi-landmarks with reference to corresponding points in a target shape, often the mean shape, which may lead to changes in the local geometric relationships among neighboring points that deviate from those in the original shape [28]. The bending energy minimization approach is also the most commonly applied in geometric morphometrics [29]. The final landmark configurations were then aligned using generalized Procrustes superimposition, in order to transform space coordinates into shape coordinates describing each subject’s position in shape space [30].

Size was determined after Procrustes superimposition and quantified as the natural logarithm of centroid size (ln(CS)) [31]. Centroid size (CS) is defined as the square root of the sum of squared distances of all landmarks from the centroid, which is the arithmetic mean position of all landmarks in the configuration [32]. The logarithmic transformation of CS is a standard practice in geometric morphometrics, ensuring isotropic variation in size-and-shape space and improving the normality of the data distribution [31, 32].

### Statistical analysis

All statistical analyses were performed using IBM SPSS statistics for Windows, Version 31.0 (IBM Corp, Armonk, NY, USA). A two-sided significance level of 0.05 was applied.

Descriptive statistics and frequency distributions were computed to summarize sample characteristics and study variables. Four multiple linear regression analyses (general linear model, full factorial design) were conducted to examine associations between each of the four size variables (ln(CS), namely the overall craniofacial configuration, cranial base, maxilla, and mandible, and the independent variables: sex (fixed factor), age (covariate), and the number of supernumerary teeth (ordinal covariate). Given the small number of individuals with three or four supernumerary teeth, the regression models were repeated in an exploratory manner: once by grouping these individuals together (≥ 3) and once by excluding them. LOESS (locally estimated scatterplot smoothing) regression was additionally used for exploratory visualization of the relationships between age and craniofacial size (ln[CS]) and between age and the number of supernumerary teeth, allowing assessment of potential non-linear trends without assuming a predefined functional form.

### Measurement error

Measurement error was assessed by repeating the digitization and ln(CS) calculation on 30 randomly selected radiographs (15 from the supernumerary group and 15 from the control group). Paired t-tests were used to compare the first and second measurements. No systematic error was observed for any CS variable (*P* > 0.45). The mean and absolute differences between repeated ln(CS) measurements were minimal, confirming excellent intraobserver reliability (Table 1).

**Table 1:** Measurement error

Size configurations	1st Meas. (Mean ± SD)	2nd Meas. (Mean ± SD)	*P*-value^1^	2nd – 1st Meas. (Mean ± SD)	|2nd – 1st | Meas. (Mean ± SD)
ln(CS) Entire craniofacial configuration	6.24 ± 0.06	6.25 ± 0.06	0.575	0.01 ± 0.08	0.06 ± 0.05
ln(CS) Cranial base	4.95 ± 0.05	4.96 ± 0.04	0.452	0.01 ± 0.06	0.04 ± 0.04
ln(CS) Maxilla	5.05 ± 0.06	5.05 ± 0.06	0.604	0.01 ± 0.09	0.06 ± 0.06
ln(CS) Mandible	5.33 ± 0.08	5.34 ± 0.08	0.680	0.01 ± 0.12	0.09 ± 0.08

Meas.: Measurement

^1^paired t-test, Level of significance: *p* < 0.05

## Results

Multiple regression analyses showed that age and sex were consistent predictors of craniofacial size (ln[CS]) across all configurations (*P* < 0.001), with size increasing with age and being smaller in females (Table 2). The relationship between age and size across configurations is illustrated in Additional File 1: Figure S2. In contrast, the number of supernumerary teeth had a limited effect, reaching statistical significance only for the maxilla (β = 0.011, *P* = 0.005), while no associations were observed for the cranial base, the mandible, or the entire craniofacial configuration (*P* > 0.05). Model fit was modest, with adjusted R² values ranging from 0.16 to 0.28 (Table 2).

Age-adjusted comparisons confirmed these findings (Table 3). No significant differences in craniofacial size were observed between individuals with and without supernumerary teeth in most regions. The only significant difference was a small increase in maxillary size in males (≈ 2.3%, *P* = 0.004).

**Table 2:** Multiple regressions of size on age, number of supernumerary teeth (0, 1, 2, 3, or 4), and sex (*n* = 400)

Size configurations*	Parameter	β-coefficient	95% CI		
			Lower bound	Upper bound	P-value
Cranial Base^1^	Intercept	4.929	4.918	4.941	< 0.001
	Age	0.002	0.002	0.003	< 0.001
	Number of supernumerary teeth	0.002	-0.004	0.007	0.529
	Female (male: reference)	-0.029	-0.038	-0.021	< 0.001
Maxilla^2^	Intercept	4.946	4.930	4.962	< 0.001
	Age	0.005	0.004	0.006	< 0.001
	Number of supernumerary teeth	0.011	0.003	0.019	0.005
	Female (male: reference)	-0.025	-0.037	-0.013	< 0.001
Mandible^3^	Intercept	5.251	5.234	5.267	< 0.001
	Age	0.006	0.005	0.007	< 0.001
	Number of supernumerary teeth	0.007	-0.001	0.015	0.079
	Female (male: reference)	-0.031	-0.044	-0.019	< 0.001
Entire craniofacial configuration^4^	Intercept	6.040	6.027	6.053	< 0.001
	Age	0.005	0.004	0.006	< 0.001
	Number of supernumerary teeth	0.006	0.000	0.012	0.054
	Female (male: reference)	-0.032	-0.042	-0.022	< 0.001

*ln(CS); ^1^Adjusted R^2^ =0.16, ^2^Adjusted R^2^ =0.19, ^3^Adjusted R^2^ =0.23, ^4^Adjusted R^2^ =0.28

**Table 3:** Age-adjusted skeletal sizes with and without supernumerary teeth. Size of individual configurations and of the entire facial configuration in subjects with and without supernumerary (control) teeth (*n* = 400). The values are adjusted for age, without considering the number of supernumerary teeth

Size configurations*		Control		Supernumerary teeth		P-value1	Mean difference
		ln (Cs)	mm	ln (Cs)	mm		
Cranial base	Females	4.93	138.5	4.92	136.4	0.218	NS
	Males	4.96	142.8	4.96	142.8	0.312	NS
Maxilla	Females	4.99	147.7	4.98	145.6	0.553	NS
	Males	5.00	148.6	5.03	152.0	0.004	2.3%
Mandible	Females	5.30	200.3	5.29	198.2	0.401	NS
	Males	5.32	204.9	5.33	206.9	0.236	NS
Entire craniofacial configuration	Females	6.07	433.8	6.07	433.8	0.502	NS
	Males	6.10	445.9	6.11	451.0	0.135	NS

Values refer to an individual of 12.7 years of age

^1^LSD test (no adjustment for multiple comparisons), Level of significance: *p* < 0.05

*ln(Cs): natural logarithm of centroid size, NS: non-significant at the 0.05 level

Sensitivity analyses yielded consistent results. When individuals with ≥ 3 supernumerary teeth were grouped, the number of supernumerary teeth remained significantly associated only with maxillary size (β = 0.010, *P* = 0.012), with no effects in other regions (Additional File 1: Table S1). Excluding these individuals did not materially change the findings, and no significant associations were observed (Additional File 1: Table S2). Age-adjusted comparisons similarly showed no differences, except for a small maxillary increase in males (Additional File 1: Table S3).

Location-specific analyses demonstrated differential effects by anatomical site (Table 4). In individuals with supernumerary teeth confined to the maxilla, no associations were found for maxillary or mandibular size (*P* > 0.05). In contrast, in individuals with mandibular supernumerary teeth, the number of supernumerary teeth was positively associated with both mandibular (β = 0.016, *P* = 0.009) and maxillary size (β = 0.022, *P* < 0.001).

**Table 4:** Regression analysis of size in location-specific supernumerary groups. Multiple regression of size on age, number of supernumerary teeth, and sex, performed on subsamples with supernumerary teeth only in the maxilla or only in the mandible, along with controls

Size configurations*	Parameter	β-coefficient	95% CI		
			Lower bound	Upper bound	*P*-value
Individuals with supernumerary teeth only in the maxilla (*N* = 145) and controls (*N* = 200)					
Maxilla^1^	Intercept	4.954	4.938	4.970	< 0.001
	Age	0.004	0.003	0.005	< 0.001
	Number of supernumerary teeth	0.002	-0.008	0.011	0.763
	Sex (reference: male)	-0.023	-0.036	-0.010	< 0.001
Mandible^2^	Intercept	5.260	5.243	5.276	< 0.001
	Age	0.005	0.004	0.006	< 0.001
	Number of supernumerary teeth	-0.005	-0.015	0.005	0.343
	Sex (reference: male)	-0.032	-0.045	-0.019	< 0.001
Individuals with supernumerary teeth only in the mandible (*N* = 50) and controls (*N* = 200)					
Maxilla^3^	Intercept	4.936	4.915	4.957	< 0.001
	Age	0.005	0.004	0.007	< 0.001
	Number of supernumerary teeth	0.022	0.010	0.034	< 0.001
	Sex (reference: male)	-0.019	-0.034	-0.004	0.013
Mandible^4^	Intercept	5.251	5.229	5.272	< 0.001
	Age	0.006	0.004	0.007	< 0.001
	Number of supernumerary teeth	0.016	0.004	0.028	0.009
	Sex (reference: male)	-0.024	-0.039	-0.008	0.002

* ln(Cs); ^1^Adjusted R^2^ =0.16, ^2^Adjusted R^2^ =0.23, ^3^Adjusted R^2^ =0.21, ^4^Adjusted R^2^ =020

Age-adjusted comparisons by location indicated that these effects were restricted to males (Table 5). In females, no significant differences were observed across configurations. In males, mandibular supernumerary teeth were associated with larger maxillary size (+ 4.39%, *P* < 0.001) and larger overall craniofacial size (+ 2.53%, *P* = 0.014), with a non-significant trend for increased mandibular size. No differences were observed at the cranial base.

**Table 5:** Skeletal configuration sizes by supernumerary tooth location. Size of individual configurations in subjects without supernumerary teeth (control, *n* = 200) and subjects with supernumerary teeth either in the maxilla (*n* = 145) or in the mandible (*n* = 50). The values are adjusted for age, without considering the number of supernumerary teeth

Size Configurations	Sex	Group	ln(Cs)	Size (mm)	*P*-value^1^	Size Difference to Controls
Cranial base	Female	No Supernumerary Teeth (Control)	4.934	138.93	—	—
		Supernumerary Teeth in Maxilla	4.922	137.28	0.124	NS
		Supernumerary Teeth in Mandible	4.932	138.66	0.899	NS
	Male	No Supernumerary Teeth (Control)	4.957	142.17	—	—
		Supernumerary Teeth in Maxilla	4.960	142.59	0.562	NS
		Supernumerary Teeth in Mandible	4.964	143.17	0.404	NS
Maxilla	Female	No Supernumerary Teeth (Control)	4.990	146.94	—	—
		Supernumerary Teeth in Maxilla	4.978	145.18	0.237	NS
		Supernumerary Teeth in Mandible	5.000	148.41	0.494	NS
	Male	No Supernumerary Teeth (Control)	5.003	148.86	—	—
		Supernumerary Teeth in Maxilla	5.017	150.96	0.076	NS
		Supernumerary Teeth in Mandible	5.046	155.40	< 0.001	+ 4.39%
Mandible	Female	No Supernumerary Teeth (Control)	5.299	200.14	—	—
		Supernumerary Teeth in Maxilla	5.279	196.17	0.068	NS
		Supernumerary Teeth in Mandible	5.317	203.77	0.225	NS
	Male	No Supernumerary Teeth (Control)	5.322	204.79	—	—
		Supernumerary Teeth in Maxilla	5.324	205.20	0.811	NS
		Supernumerary Teeth in Mandible	5.347	209.98	0.056	+ 2.53%
Entire craniofacial configuration	Female	No Supernumerary Teeth (Control)	6.076	435.28	—	—
		Supernumerary Teeth in Maxilla	6.063	429.66	0.123	NS
		Supernumerary Teeth in Mandible	6.088	440.54	0.265	NS
	Male	No Supernumerary Teeth (Control)	6.101	446.30	—	—
		Supernumerary Teeth in Maxilla	6.104	447.64	0.642	NS
		Supernumerary Teeth in Mandible	6.126	457.60	0.014	+ 2.53%

Values refer to an individual of 12.7 years of age

^1^LSD test (no adjustment for multiple comparisons)

*ln(Cs): natural logarithm of centroid size, NS: non-significant at the 0.05 level

Additional file 1: Figure S3 further illustrates the relationships between craniofacial size, sex, and the number of supernumerary teeth in the study sample. Across Tables 3 and 5, and S3, a consistent directional pattern was observed, with males showing slightly larger craniofacial dimensions in the supernumerary group, whereas females showed the opposite trend, irrespective of statistical significance.

Overall, age and sex were the primary determinants of craniofacial size, whereas associations with supernumerary teeth were limited, region-specific, and evident only in males.

## Discussion

This study was based on the hypothesis that if tooth agenesis is associated with reduced facial dimensions [17–19], the opposite anomaly, supernumerary tooth formation, may be associated with increased craniofacial size. This hypothesis is grounded in developmental biology, as dental and craniofacial tissues share common regulatory pathways and signaling mechanisms during early embryogenesis, suggesting coordinated growth and patterning processes [33, 34]. Previously reported associations between craniofacial size and tooth agenesis have been partly attributed to long-term, evolutionarily driven craniofacial adaptations, characterized by a progressive reduction in tooth number, tooth size, and facial dimensions over human evolutionary history [17, 35, 36]. Supernumerary tooth formation appears to contrast these evolutionary trends. Therefore, a weak or absent association between supernumerary tooth formation and craniofacial size would provide further support for the hypothesis that evolutionary pressures favoring fewer teeth and reduced facial dimensions remain active in modern humans. Under this framework, supernumerary tooth formation may represent a localized form of biological variation rather than a manifestation of broader craniofacial patterning changes.

Across all analyses, age and sex emerged as consistent predictors of craniofacial size. Craniofacial dimensions increased with age, reflecting normal growth and maturation, and were consistently smaller in females than in males [37, 38]. These findings underscore the necessity of age and sex adjustment when assessing size-related craniofacial outcomes [39, 40].

Overall, the associations between supernumerary teeth and craniofacial morphology were not indicative of a generalized enlargement. When the anatomical location of supernumerary teeth was considered, significant associations of the size of the entire craniofacial configuration and primarily the maxilla, were detected solely for supernumerary teeth in the mandible and only in males. There was a similar tendency for the mandible, which, however, was less pronounced and did not reach significance. No corresponding craniofacial size differences were observed in females. This sex-specific response may reflect differences in growth potential, biomechanical loading, or sex-related genetic and hormonal modulation of craniofacial development [37, 41–43].

A consistent sex-specific directional pattern was observed, with males showing slightly larger craniofacial configurations in the supernumerary group, whereas females exhibited the opposite trend, irrespective of statistical significance. This divergence may partly reflect sex differences in phenotype distribution. Males were more strongly represented and exhibited a modestly higher number of supernumerary teeth per individual (male-to-female ratio 1.32/1.16). Although mandibular supernumeraries were proportionally more frequent in females (23/75; 30.7%) than in males (32/168; 19%), a more severe mandibular phenotype (> 2 supernumerary teeth) was slightly more common in males (13/168; 7.7%) than in females (4/75; 5.3%). While these subgroup differences were exploratory and not formally tested, they may partly contribute to the more evident skeletal associations observed in males. Biological factors may also play a role, as males exhibit greater craniofacial growth magnitude and longer growth duration, potentially amplifying localized developmental effects. Given the modest effect sizes and exploratory nature of these observations, these interpretations remain cautious and warrant confirmation in larger, sex-stratified cohorts.

In contrast to the present findings, previous studies using comparable methodologies have consistently reported reduced facial dimensions in individuals with tooth agenesis. Previous studies have documented shorter maxillary length, a less convex profile, as well as a shorter face and overall smaller facial size in individuals with tooth agenesis [17, 18, 21, 44]. In addition, tooth agenesis has been associated with delayed dental development [45] and reduced dentoalveolar dimensions, further supporting its broader impact on craniofacial growth, alongside more localized effects [46].

Several conceptual frameworks may account for why tooth agenesis appears to be associated with stronger and more widespread craniofacial effects than supernumerary teeth. According to the directional developmental pressure hypothesis, tooth absence may reduce morpho-functional stimulation of the dental arches and alveolar processes, resulting in diminished skeletal growth, whereas tooth duplication may add relatively little stimulation beyond an upper functional or biological limit [47]. From an evolutionary reduction perspective, modern humans are undergoing long-term dental simplification; tooth agenesis aligns with this trajectory and may therefore engage deeply entrenched developmental pathways, producing larger phenotypic consequences. In contrast, supernumerary teeth represents a deviation from the prevailing evolutionary trend and may consequently be buffered or developmentally constrained [17, 48, 49].

From a functional perspective, tooth agenesis may reduce occlusal loading and alveolar growth, producing broader skeletal effects, whereas supernumerary teeth may not proportionally increase masticatory forces because occlusion saturates functionally and only a limited number of teeth are effectively engaged during mastication [50]. The relatively strong association between isolated third molar agenesis and facial morphology [21, 36] argues against a purely functional mechanism operating within a single lifespan, since third molars typically contribute little to occlusion, and instead points to influences acting over evolutionary timescales. Soft tissue profile findings also indicate a global influence of tooth agenesis on soft tissue profile morphology, independent of the location of dental absence [51]. Morphogen gradient theories further suggest that agenesis may disrupt signaling fields (e.g., FGF, BMP, WNT) across wider craniofacial areas, whereas supernumerary formation is more likely to induce localized changes [52, 53]. Together, these frameworks provide a developmental and evolutionary explanation for the different phenotypic impact of agenesis and supernumerary teeth, supporting the view that tooth number reductions exert broader craniofacial consequences than increases.

An additional explanation relates to developmental timing. Agenesis is often interpreted as reflecting early disturbances in odontogenesis that affect the dental lamina or bud stages, which coincide with critical periods of craniofacial patterning. Disruptions during these early windows may therefore propagate across multiple craniofacial structures [54, 55]. In contrast, supernumerary formation is commonly attributed to localized dental lamina hyperactivity or splitting processes that exert more regionally confined effects and occur within an already established developmental framework, limiting their capacity to induce widespread facial change [55]. Consistent with this view, non-syndromic agenesis is associated with delayed dental development, whereas non-syndromic supernumerary formation shows dental maturation rates comparable to unaffected children [55].

Differential skeletal responses to mechanical loading may further contribute to this disproportion. According to the mechanostat theory, reduced mechanical input promotes bone resorption, whereas increased loading above physiological thresholds stimulates bone formation [56]. In agenesis, decreased functional loading of the alveolar processes may amplify skeletal reduction, whereas supernumerary teeth are unlikely to increase loading beyond normal functional levels and thereby stimulate enhanced bone formation. These mechanisms likely operate over evolutionary rather than short developmental timescales, as evidenced by the strong association between third molar agenesis and facial form despite minimal functional input [21, 36], and by the lack of vertical facial differences between extraction and non-extraction orthodontic treatment [57]. Together, these observations support the view that agenesis and supernumerary teeth represent opposing outcomes along a shared developmental continuum influencing craniofacial growth.

From an evolutionary perspective, variation in tooth number aligns with broader trends of dental reduction in modern humans. Agenesis reflects this long-term simplification, whereas supernumerary teeth may represent localized developmental instability or persistence of ancestral variation. The predominantly regional effects observed here, particularly for mandibular supernumerary teeth, likely reflect region-specific developmental sensitivity rather than a global craniofacial response. Because mandibular supernumeraries represent a relatively rare phenotype (approximately 1:2.4 compared with maxillary cases) [1] and a developmentally more extreme variant, their effects should not be assumed to generalize to supernumerary tooth formation overall.

Clinically, the present findings suggest that supernumerary teeth are unlikely to be associated with meaningful craniofacial size alterations, except for minor regional enlargements, predominantly in males and when supernumerary teeth are located in the mandible. Although statistically significant, these effects are small in magnitude and should be individually assessed in treatment planning.

The present study was conducted in a well-defined White European cohort, allowing for reduced population heterogeneity and more precise estimation of craniofacial size relationships within this group. While certain subtypes of supernumerary teeth were less frequent, the overall sample size was sufficient to detect consistent and clinically meaningful patterns. A limitation is that supernumerary teeth were classified only according to jaw location (maxilla or mandible). More detailed anatomical characteristics, including anterior versus posterior position, eruption status, morphology, and potential involvement in occlusion, may also influence craniofacial development. Because the frequencies of specific supernumerary tooth subtypes were low, particularly within the mandibular group, meaningful stratified analyses were not feasible. Therefore, the present findings should be interpreted primarily in relation to jaw location, while acknowledging that future studies with larger samples could further explore the potential influence of more specific supernumerary tooth characteristics. Inclusion of growing individuals reflects the real-world clinical population and supports the stability of the observed associations across developmental stages. Although analyses were based on two-dimensional cephalometric radiographs, these measures capture established craniofacial dimensions that are robust and widely validated, and the observed relationships are unlikely to be altered by three-dimensional imaging. The moderate explanatory power of the regression models further suggests that craniofacial size is influenced by multiple interacting biological and environmental factors, consistent with its multifactorial nature.

## Conclusion

Supernumerary teeth are associated with limited and region-specific craniofacial effects rather than generalized facial enlargement. A greater number of supernumerary teeth was linked to a small increase in maxillary size, and mandibular supernumeraries were associated with modest enlargement of both jaws, predominantly in males. No consistent overall craniofacial size changes were detected.

These findings differ from the well-established associations between tooth agenesis and reduced facial dimensions reported in previous studies using comparable methodologies, suggesting that different tooth-number anomalies may be associated with distinct patterns of craniofacial development. Supernumerary teeth likely reflect localized developmental variation within an evolutionary framework that favors craniofacial reduction, resulting in limited skeletal consequences.

## Supplementary Information

Supplementary Material 1.

## Data Availability

The protocols and datasets generated and/or analysed during the current study are available from the corresponding author on reasonable request.

## Abbreviations

ANS: Anterior nasal spine
APC: Adenomatous polyposis coli
BMP: Bone morphogenetic protein
CBCT: Cone-beam computed tomography
CI: Confidence interval
CS: Centroid size
EDA: Ectodysplasin A
FGF: Fibroblast growth factor
FAP: Familial adenomatous polyposis
ln(CS): Natural logarithm of centroid size
LOESS: Locally estimated scatterplot smoothing
OMIM: Online Mendelian Inheritance in Man
PNS: Posterior nasal spine
R²: Coefficient of determination
SHH: Sonic hedgehog
SPSS: Statistical Package for the Social Sciences
WNT: Wingless/Integrated signaling pathway
