# Supplementary material for: Craniofacial size in individuals with supernumerary teeth: A multicenter geometric morphometric study
Source: BMC Oral Health. 2026 Jul 7;26:1870. doi: 10.1186/s12903-026-09159-9 (PMC13625379; doi:10.1186/s12903-026-09159-9)
Supplement: Supplementary file 1 [file 12903_2026_9159_MOESM1_ESM.docx]

*BMC Oral Health*

*Additional File 1*

**Craniofacial size in individuals with supernumerary teeth: A multicenter geometric morphometric study**

Sofia Bakirtzi, Eva Henninger, Luca Friedli, Georgios Kanavakis, Nikolaos Gkantidis

**Table S1. Multiple regressions of size on age, number of supernumerary teeth (0, 1, 2, or ≥3), and sex (n=400).**

| Size configurations* | Parameter | β-coefficient | 95% CI | | |
| --- | --- | --- | --- | --- | --- |
|  |  |  | Lower bound | Upper bound | P-value |
| Cranial Base^1^ | Intercept | 4.930 | 4.919 | 4.941 | <0.001 |
|  | Age | 0.002 | 0.002 | 0.003 | <0.001 |
|  | Number of supernumerary teeth | 0.001 | -0.004 | 0.007 | 0.670 |
|  | Female (male: reference) | -0.029 | -0.038 | -0.021 | <0.001 |
| Maxilla^2^ | Intercept | 4.946 | 4.930 | 4.962 | <0.001 |
|  | Age | 0.005 | 0.004 | 0.006 | <0.001 |
|  | Number of supernumerary teeth | 0.010 | 0.002 | 0.019 | 0.012 |
|  | Female (male: reference) | -0.026 | -0.038 | -0.014 | <0.001 |
| Mandible^3^ | Intercept | 5.252 | 5.235 | 5.268 | <0.001 |
|  | Age | 0.006 | 0.005 | 0.007 | <0.001 |
|  | Number of supernumerary teeth | 0.006 | -0.002 | 0.015 | 0.156 |
|  | Female (male: reference) | -0.032 | -0.044 | -0.019 | <0.001 |
| Entire craniofacial configuration^4^ | Intercept | 6.041 | 6.028 | 6.054 | <0.001 |
|  | Age | 0.005 | 0.004 | 0.006 | <0.001 |
|  | Number of supernumerary teeth | 0.005 | -0.001 | 0.012 | 0.115 |
|  | Female (male: reference) | -0.032 | -0.042 | -0.023 | <0.001 |

*ln(CS); ^1^Adjusted R^2^ =0.15, ^2^Adjusted R^2^ =0.19, ^3^Adjusted R^2^ =0.22, ^4^Adjusted R^2^ =028.

**Table S2. Multiple regressions of size on age, number of supernumerary teeth (0, 1, or 2) and sex (n=393).**

| Size configurations* | Parameter | β-coefficient | 95% CI | | |
| --- | --- | --- | --- | --- | --- |
|  |  |  | Lower bound | Upper bound | P-value |
| Cranial Base^1^ | Intercept | 4.931 | 4.920 | 4.942 | <0.001 |
|  | Age | 0.002 | 0.001 | 0.003 | <0.001 |
|  | Number of supernumerary teeth | -0.001 | -0.008 | 0.005 | 0.687 |
|  | Female (male: reference) | -0.029 | -0.037 | -0.020 | <0.001 |
| Maxilla^2^ | Intercept | 4.948 | 4.932 | 4.964 | <0.001 |
|  | Age | 0.005 | 0.004 | 0.006 | <0.001 |
|  | Number of supernumerary teeth | 0.008 | -0.001 | 0.017 | 0.087 |
|  | Female (male: reference) | -0.025 | -0.037 | -0.013 | <0.001 |
| Mandible^3^ | Intercept | 5.255 | 5.238 | 5.271 | <0.001 |
|  | Age | 0.006 | 0.004 | 0.007 | <0.001 |
|  | Number of supernumerary teeth | 0.002 | -0.008 | 0.011 | 0.729 |
|  | Female (male: reference) | -0.030 | -0.043 | -0.018 | <0.001 |
| Entire craniofacial configuration^4^ | Intercept | 6.043 | 6.030 | 6.056 | <0.001 |
|  | Age | 0.005 | 0.004 | 0.006 | <0.001 |
|  | Number of supernumerary teeth | 0.002 | -0.005 | 0.010 | 0.536 |
|  | Female (male: reference) | -0.031 | -0.041 | -0.022 | <0.001 |

*ln(CS); ^1^Adjusted R^2^ =0.15, ^2^Adjusted R^2^ =0.18, ^3^Adjusted R^2^ =0.22, ^4^Adjusted R^2^ =027.

**Table S3. Age-adjusted skeletal sizes excluding three or four supernumerary teeth.** Size of individual configurations and of the entire facial configuration in subjects with and without supernumerary (control) teeth, excluding individuals with three or four supernumerary teeth (n=393). The values are adjusted for age.

| Size configurations* | | Control | | Supernumerary teeth | | P-value | Mean difference |
| --- | --- | --- | --- | --- | --- | --- | --- |
|  |  | ln (Cs) | mm | ln (Cs) | mm |  |  |
| Cranial base | Females | 4.93 | 138.5 | 4.92 | 136.4 | 0.217 | NS |
|  | Males | 4.96 | 142.8 | 4.96 | 142.8 | 0.470 | NS |
| Maxilla | Females | 4.99 | 147.7 | 4.98 | 145.6 | 0.551 | NS |
|  | Males | 5.00 | 148.6 | 5.02 | 151.6 | 0.010 | 2% |
| Mandible | Females | 5.30 | 200.3 | 5.29 | 198.2 | 0.394 | NS |
|  | Males | 5.32 | 204.9 | 5.33 | 206.9 | 0.434 | NS |
| Entire craniofacial configuration | Females | 6.08 | 436.5 | 6.07 | 433.8 | 0.497 | NS |
|  | Males | 6.10 | 445.9 | 6.11 | 451.0 | 0.258 | NS |

Values refer to an individual of 12.7 years of age.

*ln(Cs): natural logarithm of centroid size, NS: non-significant

**Figure S1**. **Sample distribution according to age, sex and number of supernumerary teeth.** The colored lines represent LOESS (Locally Estimated Scatterplot Smoothing) best fit lines per sexual subgroup.

**Figure S2. Scatterplots displaying size variability according to age.** The colored lines represent LOESS (Locally Estimated Scatterplot Smoothing) best fit lines per sexual subgroup. ln(CS): natural logarithm of centroid size

**Figure S3**. **Box plots showing size variability per sex, according to the number of supernumerary teeth.** Each plot refers to a specific anatomical configuration denoted in the respected y-axis legend. ln(CS): natural logarithm of centroid size
